# A Wireless Multi-Sensor Dielectric Impedance Spectroscopy Platform

**DOI:** 10.3390/s150923572

**Published:** 2015-09-17

**Authors:** Seyed Alireza Ghaffari, William-O. Caron, Mathilde Loubier, Maxime Rioux, Jeff Viens, Benoit Gosselin, Younes Messaddeq

**Affiliations:** 1 Department of Electrical and Computer Engineering, Laval University, Quebec, QC G1V 0A6, Canada; E-Mails: seyed-alireza.ghaffari.1@ulaval.ca (S.A.G.); Benoit.Gosselin@gel.ulaval.ca (B.G.); 2 Department of Chemistry, Laval University, Quebec, QC G1V 0A6, Canada; E-Mails: william-olivier.caron.1@ulaval.ca (W.-O.C.); mathilde.loubier.1@ulaval.ca (M.L.); maxime.rioux.2@ulaval.ca (M.R.); younes.messaddeq@copl.ulaval.ca (Y.M.); 3 Centre for Optics, Photonics and Lasers (COPL), Laval University, Quebec, QC G1V 0A6, Canada

**Keywords:** dielectric impedance spectroscopy, electrochemical impedance spectroscopy, ZigBee Mesh, wireless network

## Abstract

This paper describes the development of a low-cost, miniaturized, multiplexed, and connected platform for dielectric impedance spectroscopy (DIS), designed for in situ measurements and adapted to wireless network architectures. The platform has been tested and used as a DIS sensor node on ZigBee mesh and was able to interface up to three DIS sensors at the same time and relay the information through the network for data analysis and storage. The system is built from low-cost commercial microelectronics components, performs dielectric spectroscopy ranging from 5 kHz to 100 kHz, and benefits from an on-the-fly calibration system that makes sensor calibration easy. The paper describes the microelectronics design, the Nyquist impedance response, the measurement sensitivity and accuracy, and the testing of the platform for in situ dielectric impedance spectroscopy applications pertaining to fertilizer sensing, water quality sensing, and touch sensing.

## 1. Introduction

The evolutionary progress of information and communications technology is enabling continuous improvements on in situ sensor technology. Internet connectivity allows physical objects (e.g., household goods, industrial equipment, food and agriculture settings, *etc.*) to be sensed and controlled remotely across existing network infrastructure such that embedded computing system can collect up-to-date information on objects and processes, creating opportunities for more direct integration between in situ measurements and computer-based systems [1]. In this scenario, often coined the Internet of Things (IoT), objects are provided with identifiers and with the ability to transfer data over a network without requiring human interaction. This enables many aspects of everyday objects to be monitored in situ at a previously unattained level of detail and be interoperable at low cost wirelessly within the existing Internet infrastructure. The ability to sense and react to events in the physical world in an automatic, rapid, and informed manner not only opens up new opportunities for dealing with complex or critical situations, but also enables a wide variety of processes to be better understood and optimized cost-effectively [2].

**Figure 1:**
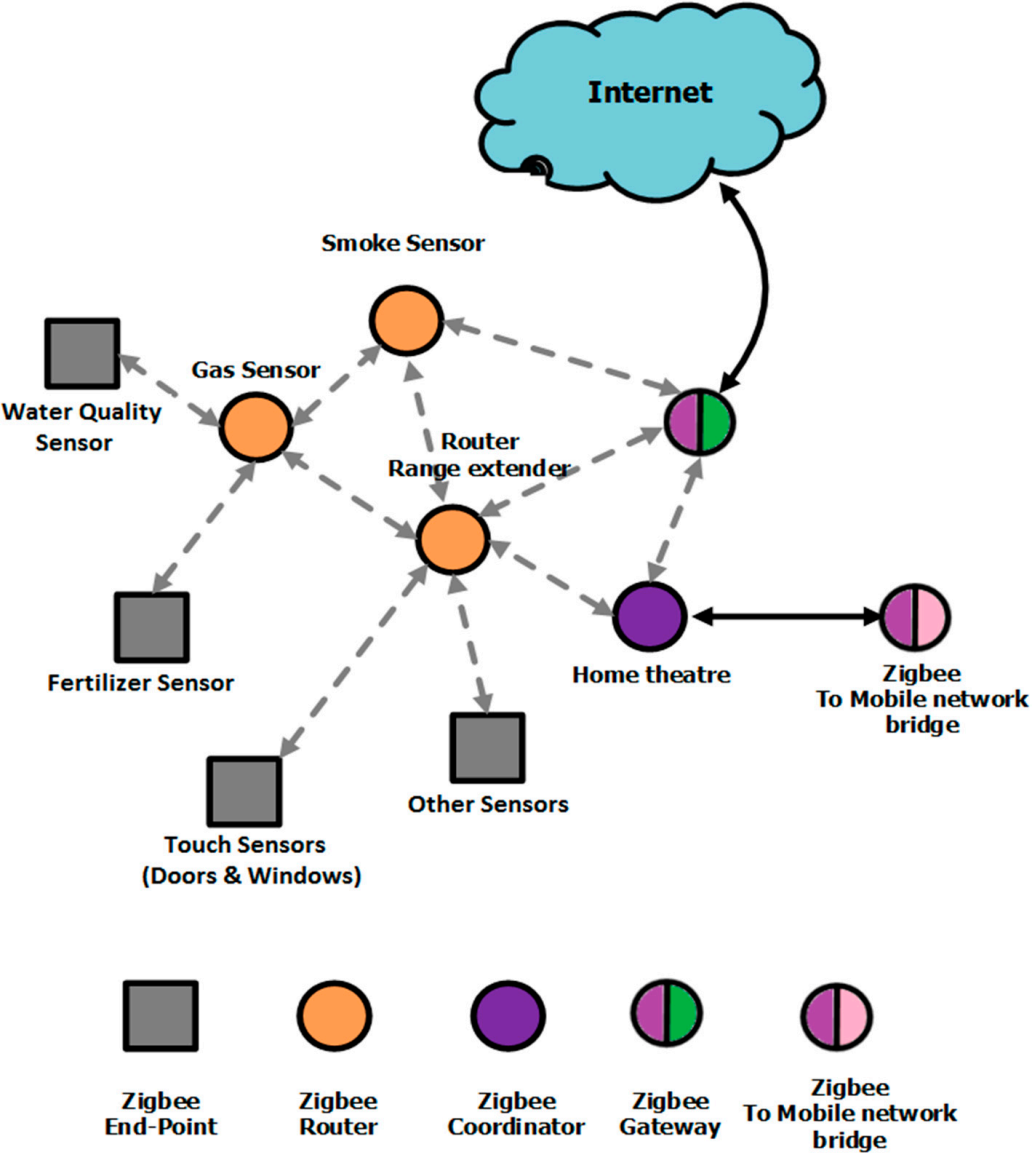
Network Architecture for smart homes [3] using a ZigBee platform that can be adapted to the connectivity of DIS and EIS sensor devices, such as water quality sensors, fertilizer sensors, and touch sensors.

The adoption of connected in situ sensors is largely driven by opportunities to increase productivity, cost-efficiency, monitoring and control of assets [3]. Sensor devices based on dielectric impedance spectroscopy (DIS) or electro-chemical impedance spectroscopy (EIS) exhibit compatibility to IoT architectures to the extent that their design can offer low-cost, miniaturized, multiplexed, and connected functionalities, which are key attributes to defining smart strategies for efficient technology scaling and sustainable growth [4]. Network connectivity of DIS sensor devices is a core function for future service and application development; thanks to the rapid development of microelectronics technology, the cost of wireless network connectivity is no longer a significant factor. Instead of giving DIS sensor devices conventional standalone operating controls and displays, it becomes progressively more cost-effective to fit them with a wireless interface, such as ZigBee, and export their interaction components to the Internet or cellular networks. Of particular interest are the sensor platforms addressing IEEE’s 802.15.4 protocol, enabling low-power low-rate wireless personal area networks (LR-WPANs) for the use of 2.4 GHz radio frequency (RF) channels [5]. The interconnection of embedded DIS platforms onto ZigBee meshes is expected to usher in automation in nearly all fields, enabling advanced applications like Smart homes, Smart Grid, and Smart city [3] in order to interface multiple end-point sensors to control and monitor a range of assets. To illustrate such sensor connectivity to Smart homes, Figure 1 depicts a ZigBee wireless network architecture that can be adapted to the connectivity of DIS and EIS sensor devices, which may include sensing end-points such as water quality sensors, fertilizer sensors, and touch sensors, *etc.*, that find applications in many smart household or building environments.

This paper describes a low-cost, miniaturized, multiplexed, and connected platform for continuous-frequency dielectric impedance spectroscopy (DIS) and electro-chemical impedance spectroscopy (EIS) designed for in situ measurements and adapted to wireless network architectures. The DIS platform was built from commercial off-the-shelf microelectronics components comprising a calibration multiplexer, a dielectric spectroscopy analyser ranging from 5 kHz to 100 kHz frequency with a resolution of 0.2 Hz, a digital signal controller, and a ZigBee transceiver. The platform has been tested for in situ measurement applications pertaining to fertilizer sensing, water quality sensing, and touch sensing, and the concept can be scaled cost-effectively to much larger networks of dielectric impedance sensors.

## 2. Design description of the Wireless DIS Platform

### 2.1. System Overview

**Figure 2:**
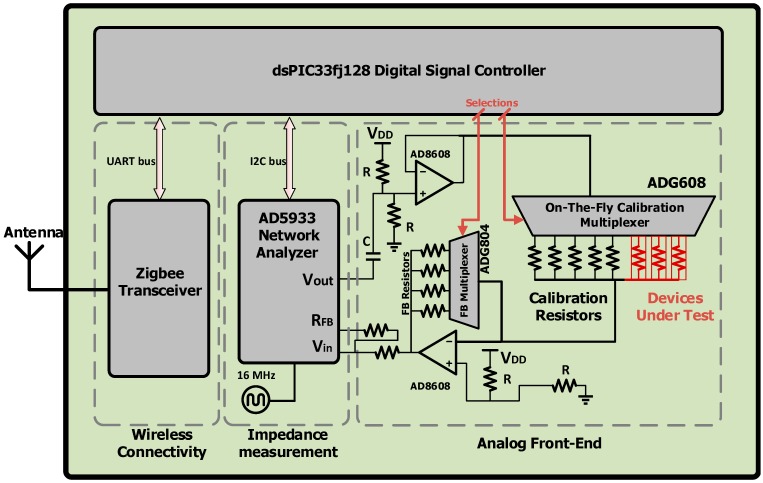
Functional block diagram of the dielectric impedance spectroscopy (DIS) platform.

The developed dielectric impedance spectroscopy platform consists of three main building blocks: (1) an on-the fly calibration and digitally controlled feedback loop; (2) a wireless connectivity module consisting of a ZigBee Mesh; and (3) a continuous-frequency dielectric impedance spectroscopy unit. This three-block architecture provides the interface that links in situ DIS sensors to wireless networks. The three blocks are connected to a 16-bit digital signal controller [6] that carries out the command/data handling and impedance measurement for this system. Figure 2 schematizes the functional block diagram of the DIS platform.

The first block is an analog front end for setting the DC level of the excitation voltage of the sensors. On-the-fly calibration is a microelectronics functionality that allows DIS and EIS sensor devices to be multiplexed to the same microelectronics platform, thus providing the advantage of monitoring multiple sensors at the same time using the same microelectronics board. The user can calibrate the sensor devices for different impedance measurements ranges with known on-board impedances. On-the-fly calibration and digitally controllable trans-impedance amplifiers feedback loop give the ability to connect multiple different sensors to the device, while digitally controlled feedback loop reaches suitable gain-factor for internal trans-impedance amplifiers. For the DIS platform developed in this work, the digitally controlled front-end is designed to multiplex over three different DIS sensors labeled as Devices Under Test (DUT) with five calibration/etalon resistors, all connected to a 8:1 multiplexer. This circuit allows on-the-fly switching between the sensors, and the different calibration resistors prevent saturation of the AD5933 dielectric impedance measurement unit.

The second block consists of a wireless connectivity through a ZigBee Mesh consisting of a commercial XBee radio module [7] from Digi International, designed for point-to-point communications at data rates of 250 kbit/s.

The third block is a dielectric impedance measurement unit comprising an AD5933 precision impedance converter circuit from Analog Devices that uses an auto-balancing method [8,9] for dielectric impedance measurements up to 10 MΩ, with a 12-bit analog-to-digital converter (ADC). The frequency generator allows an external complex impedance (*i.e.*, sensor) to be excited with a known AC frequency. The impedance response signal is sampled by the ADC and a discrete Fourier transform (DFT) is then processed by the on-board engine [10]. The continuous-frequency sweep of the AD5933 device provides for much higher informational value than multi-frequency based sensing, to the extent that higher frequency resolution provides for better modeling and understanding of the DUT dielectric response*.*

**Figure 3:**
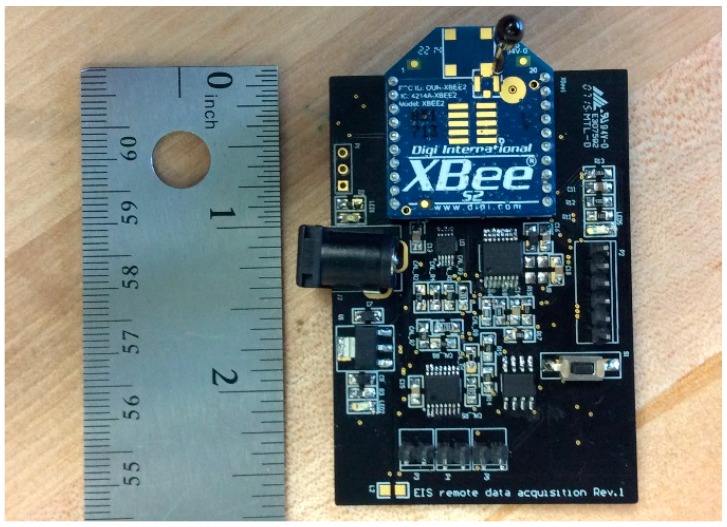
Picture of the wireless Dielectric Impedance Spectroscopy (DIS) Platform.

The DIS platform comprises two multiplexer components, the ADG804 and ADG608, which are used for feedback multiplexer and on-the-fly calibration multiplexer, respectively. These two multiplexers allow the user to change the range of impedance measurement through a ZigBee network. The user can calibrate the platform with a different range of impedances, and even measure three sensors while using just one calibration resistor. This multiplexing technique helps reduce the overall cost of the platform, and can be scaled to multiplexing values much larger than the 8:1 figure chosen in this work. Figure 3 shows an actual picture of the wireless DIS platform. This platform weighs only 18 grams, exhibits small dimensions (4 cm × 6 cm), and costs less than 90 USD.

### 2.2. Dielectric Impedance Measurement Unit

The dielectric impedance measurement unit consists of the AD5933 network analyzer device and 16 MHz external clock for its internal Direct Digital Synthesis (DDS) engine. The DDS engine produces a programmable frequency sine wave as the excitation wave for the DIS sensors (*i.e.*, DUT). The DDS is capable of exciting the DUT at different voltage levels, wherein the DC voltage level (*i.e.*, DC bias) is set at the front-end and the AC voltage level (*i.e.*, AC amplitude) is programmable to values of 200 mV, 400 mV, 1 V, and 2 V. An AC amplitude of 200 mV and a DC bias of less than 1 V were preferred as higher values led to a non-linear response, while lower AC values led to poor S/N response and poor measurement accuracy. The AD5933 employs a trans-impedance amplifier for amplifying the current and then uses the 1024-point on-chip DFT algorithm for separating the real and imaginary parts of the impedance. The AD5933 range of frequency for dielectric impedance measurements is limited from 5 kHz to 100 kHz, with a resolution of 0.2 Hz, according to restrictions determined by the 16 MHz oscillator module linked to the AD5933 device [11]. The AD5933 network analyzer device is calibrated with etalon resistors, exhibiting resistance values similar to the impedance range of the DIS sensors. For example, if the DIS sensor provides dielectric impedances ranging from 500 Ω to 1500 Ω, then the ideal calibration resistor would be a 1-kΩ load. The calibration should be done with purely resistive loads in order to determine the internal AD5933 phase. AD5933 does have the phase offset due to internal zeros and poles of the DDS engine, therefore calibration with a known resistor helps to extract this phenomenon.

### 2.3. Wireless Connectivity through ZigBee Mesh

The DIS platform is equipped with a ZigBee communication transceiver capable of being used within a ZigBee Mesh network having thousands of nodes, thus providing significant DIS scaling capabilities. As shown previously in Figure 1, the ZigBee mesh is composed of three main categories of nodes: ZigBee End Points, ZigBee Coordinators, and ZigBee Routers. ZigBee End Points correspond to specific DIS sensors; these nodes communicate with their parent router nodes, and are able to be switched into a low-energy consumption state (*i.e.*, a sleep mode). ZigBee Coordinator Nodes control the formation and the security of the networks, whereas ZigBee routers extend the range toward the broader communication network. Routers are usually multi-function nodes that can be reserved as a sensor node and still route the data to other nodes. The DIS platform described in this work can perform all three functionalities of the ZigBee nodes by loading the suitable firmware on its ZigBee transceiver.

### 2.4. Digital Signal Controller

**Figure 4:**
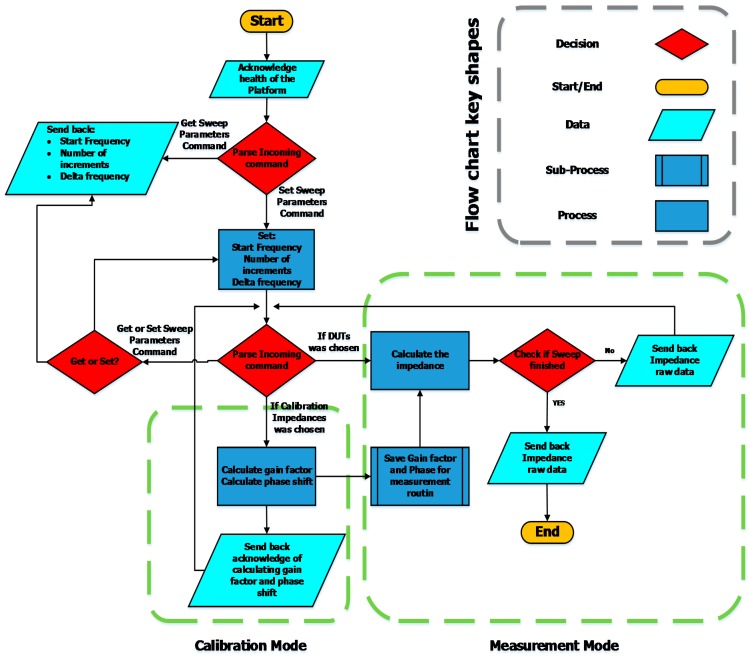
Flowchart of the DIS platform embedded firmware.

All the command and data handling functionality of the analog front-end, impedance measurement unit, and ZigBee transceiver are accomplished by an on-board 16-bit micro-controller unit (MCU), shown in Figure 2 [6]. The dsPIC33fj128 MCU communicates through I2C bus with impedance measurement unit and employs a universal asynchronous receiver/transmitter (UART) to communicate with the ZigBee transceiver. The microcontroller firmware sends the appropriate command to the different units of the platform, collects the data, and sends it back through the ZigBee network to the superior nodes. The firmware is designed to provide all the functionalities of the board, such as calibration setting, voltage level setting, frequency and sweep parameters setting, and calibration resistor setting. The board has the ability to be calibrated with on-board resistors as the device gets the command of the calibration through the Zigbee network, removing the need to connect the device to a computer. The MCU unit sends back the raw data of the impedance and gain factor at each stage of the calibration command and acknowledges the user of healthy functionality of the board. Figure 4 shows the flowchart of the DIS platform embedded firmware.

The stability of the DIS platform signal *versus* time is invariant due to nature of the digital design; the time stability of the signal is predominantly limited by the material/environment/temperature stability of the sensors connected to the DIS platform. The platform’s power consumption depends on the functionality of the nodes in the ZigBee mesh. The ZigBee module is the most power-hungry part of the board, drawing 75 mA electrical current during data acquisition (50 mA of which is attributable to the wireless communication functionality), which represents a 250-mW power consumption at 3.3-Volt driving potential. A single data acquisition is usually performed in less than 2 s, after that the board will draw around 25 mA of current in stand-by mode. The platform may be switched to a power-down mode at the end-points of the ZigBee mesh with a current consumption of less than 5 mA.

## 3. Experimental Testing of the DIS Platform for in Situ Dielectric Impedance Spectroscopy

This low-cost, miniaturized, multiplexed, and connected platform has been tested and used experimentally as a DIS sensor node on ZigBee mesh. The platform interfaced three DIS sensors at the same time and relayed the information through the Zigbee network for data analysis and storage. Specifically, the platform has been tested for in situ dielectric impedance spectroscopy applications pertaining to fertilizer sensing, touch sensing, and water quality sensing, that find applications in many smart household, building, and industrial environments. To our knowledge, this is the first time a wireless platform, featuring an AD5933 device, was used along with touch and chemical sensors for environmental monitoring. For the sake of care, this warranted comparison with other known and well-calibrated impedance meters, such as the Solartron 1260 Impedance Analyzer [12], which provides accurate complex impedance measurements in the AC frequency range from 1 Hz to 1 MHz.

### 3.1. Electro-Chemical Impedance Spectroscopy (EIS) Nitrate Sensors

Fertilizer sensors, such as nitrate sensors, controlled by DIS platforms find many applications in agriculture and allow the feeding of sensor data into online databases for continuous crop monitoring, production optimization, and data storage. A research challenge in this field is focused on the need to develop rapid, reliable, specific, and sensitive methods to detect and monitor these nutrients cost-effectively [13,14], while large scale analysis implies improved miniaturization, reduction of analysis time and cost, and wireless connectivity [15].

**Figure 5:**
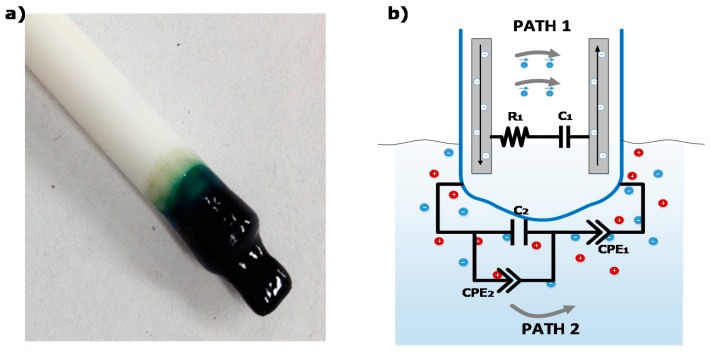
(**a**) Picture of the EIS nitrate sensor head connected to the DIS platform; and (**b**) Schematics of the main electrical conduction paths of the EIS nitrate sensor: one through the polymer membrane and the other throughout the medium under test, with corresponding equivalent electrical circuits [4].

The tested nitrate sensor [4] comprises a set of electrode wires surrounded by an ion selective polymer membrane, as shown in Figure 5a, and is controlled by the DIS platform described previously. The polymer membrane is inserted into the medium (preferably wet) and interacts locally with the medium under test. This sensor configuration provides two main electrical conduction paths, one within the polymer membrane, and the other into the medium under test, depicted as paths 1 and 2 in Figure 5b, respectively. It is worth mentioning that both the membrane (path 1) and medium (path 2) are predominantly dielectric materials in nature and therefore exhibited poor DC electrical conductivities. Their electrical properties relate to various delocalized electrical carrier conduction mechanisms at play in non-crystalline materials, such as short-range carrier mobility, defect hopping/trapping, and carrier diffusion, which manifest themselves negligibly in DC but may become significant and easily-measured at AC frequencies in the kHz and MHz range [4]. Therefore, electrical conductivity measurements of highly resistive materials becomes possible at high AC frequencies, thus bringing the range of measurability within the 10-MΩ impedance sensing limit of the AD5933 device. In this context, the AD5933 device was designed with a 16 MHz oscillator providing the highest frequency range of 5 kHz to 100 kHz [11], and the equivalent electrical circuit model contained both resistance and reactance (capacitive) elements, which governed carrier mobility at this driving frequency range. Specifically, the electrical circuit model assigned resistances and capacitances related to the nitrate-selective polymer membrane (Path 1: R_1_, C_1_) and to the non-selective medium under test (Path 2: C_2_-CPE_1_-CPE_2_), which comprised phenomenological constant phase elements (CPE) that account for carrier diffusion mechanisms and electrical double layers.

The polymer membrane is composed of high molecular weight polyvinyl chloride (PVC—from Aldrich) and of a plasticizer bis(2-ethylhexyl) phthalate (BEHP—also from Aldrich). Ion-selectivity is provided by adding two components to the polymer membrane: an ionophore and ionic sites. For the nitrate sensor, the ionophore consisted of tetramethyl cyclotetra-decanato-nickel(II) complex (NiTMTAA), and the ionic site consisted of trioctylmethylammonium chloride (TOMAC—from Aldrich). Both of these have been chosen according to the reversibility, selectivity (>4 $pK_{NO_{3}^{-},A^{-}}^{pot}$, where A^−^ stands for NO_2_^−^, HPO_4_^2−^, SO_4_^2−^, or Cl^−^) and efficiency reported in previous potentiometric studies [16]. Together, this polymer membrane composition can be dissolved into tetrahydrofurane and molded into any desired shape prior to drying, which brings mechanical strength, environmental endurance, and abrasion resistance, and which defines a stable baseline of electrical conductivity to the system.

**Figure 6:**
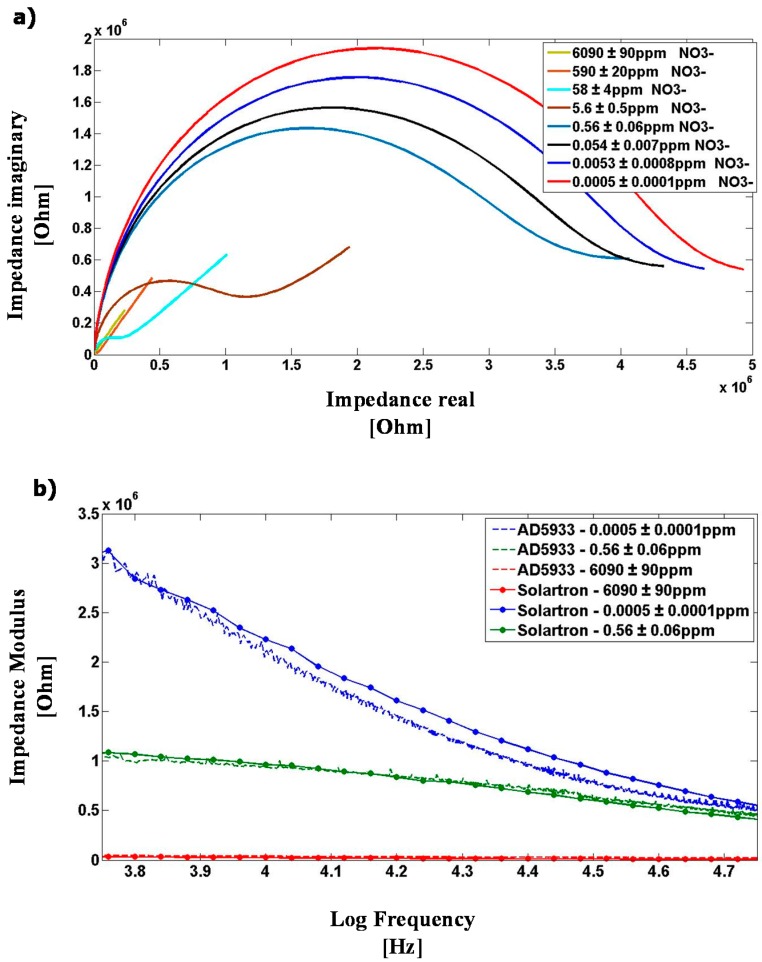
(**a**) Nyquist response of the EIS nitrate sensor as measured by the Solartron 1260 Impedance analyzer from 1 Hz to 1 MHz frequency, tested through a wide range of nitrate (NO_3_**^−^**) concentrations; and (**b**) Comparative EIS nitrate sensor measurement results obtained using the Solartron Impedance analyzer and the DIS Platform featuring an AD5933 device.

Impedance measurements were performed by immersing the EIS nitrate sensor in 10 mL of KNO_3_-containing (Aldrich—selectophore grade) deionized water (18 MΩ·cm) solutions set at 20 °C, room temperature. The sensor was left immersed in the solution for about 5 min to provide enough time for interaction and equilibrium with the ions. After this settling time, the sensor response became time invariant. Figure 6a shows the impedance spectra of the immersed EIS nitrate sensors through a wide range of nitrate (NO_3_**^−^**) concentrations, as measured by the Solartron Impedance analyzer, revealing strong dependence on nitrate concentration throughout the range from 0 to 6000 ppm. As expected, the polymer membrane exhibited a negative phase in the impedance spectra indicative of a resistance-capacitance material response (path 1), which, however, became strongly attenuated by the non-selective impedance of the medium at NO_3_**^−^** concentrations higher than 100 ppm (path 2). The response time of the sensor was measured at about 1 min when the impedance response was dominated by path 1, and about 1 s when dominated by path 2.

The Solartron impedance analyzer measurements have been compared with the DIS platform measurements under the same laboratory conditions, as shown in Figure 6b. The AD5933 device within the DIS platform exhibited higher measurement errors when operating close to upper or lower frequency limits of 5 kHz and 100 kHz, or when the sensor exhibited an impedance value far from the 200-kΩ calibration resistance. Overall, good agreement in impedance measurements was obtained between the AD5933 and Solartron, to within a ±10% comparative error level, through wide a range of nitrate concentrations. The comparative errors were about ±2% at 200 mV AC amplitude between 0.1 ppm and 100 ppm nitrate, which is the typical measurement range of nitrate sensors for agriculture applications.

### 3.2. Water Quality Sensors

In addition to being used as nitrate fertilizer sensors for agriculture, the abovementioned EIS nitrate sensors linked with the DIS platform can be used as a basis for water quality sensors, to the extent that these sensors become sensitive to a wide variety of ions in the medium under test when the conductivity of the medium (path 2) dominates the conductivity of the membrane (path 1), which occurs at ion concentrations higher than 100 ppm with the abovementioned sensor design. Water quality is a measure of the condition of water relative to the requirements of human consumption, and it is most frequently used by reference to a set of standards against which compliance can be assessed [17]. In North America, standards pertaining to the suitability of water for human consumption are usually regulated at the state level, and the following contaminant concentration limits in water may be found: Nitrates (NO_3_^−^) 45 ppm; Chloride (Cl^−^) 307 ppm; Sodium (Na^+^) 199 ppm; Sulfates (SO_4_^2−^) 560 ppm [18]. Contaminants that may be found at hundreds of ppm concentration in water include inorganic runoffs from agricultural activities (e.g., nitrate, phosphate, potassium), which are a major cause of water pollution in rural areas. The complexity of water quality as a subject is reflected in the many types of measurements of water quality indicators. Measurements commonly made on-site and in direct contact with the water source include temperature, pH, dissolved oxygen, conductivity, oxygen reduction potential (ORP), and turbidity [19]. Nowadays, citizens demand real-time information about the water they use daily and are drinking. Unfortunately, such information can be very expensive to obtain as water quality is usually sampled and analyzed at certified laboratories requiring water samples to be collected, preserved, transported, and analyzed at diverse locations.

**Figure 7:**
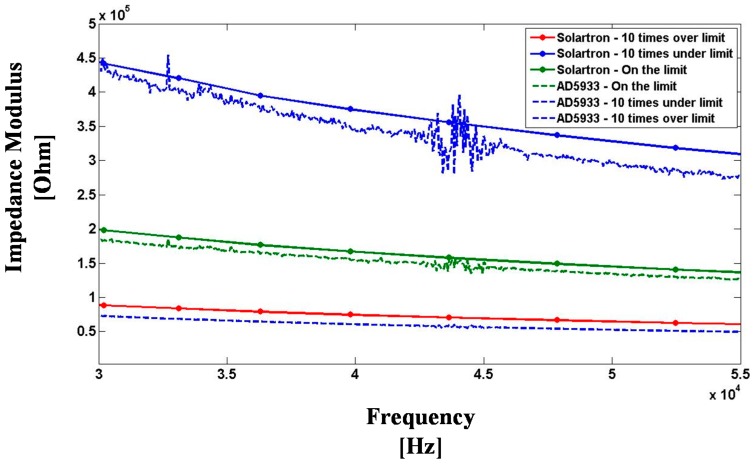
Comparative EIS water quality sensor results obtained using the Solartron Impedance analyzer (solid lines) and the DIS platform (dotted lines), wherein the baseline of 45 ppm nitrate (NO_3_^−^), 307 ppm chloride (Cl^−^), 199 ppm sodium (Na^+^), and 560 ppm sulfate (SO_4_^2−^) has been used to define the contaminant concentration limit in water.

The IS nitrate sensor connected to the DIS sensor node on ZigBee mesh provides an affordable in situ measurement technology for water quality sensing. In order to test the suitability of the DIS platform for water quality sensing, a set of water samples have been prepared with known concentrations of nitrate, chloride, sodium and sulfate contaminants. The abovementioned baseline of 45 ppm nitrate (NO_3_^−^), 307 ppm chloride (Cl^−^), 199 ppm sodium (Na^+^), and 560 ppm sulfate (SO_4_^2−^) has been used to define contaminant concentration limit in water, wherein the water samples have been prepared by mixing selected molar quantities of potassium nitrate (KNO_3_) (Sigma-Aldrich-ReagentPlus ≥99.0%), potassium sulfate (K_2_SO_4_) (Sigma-Aldrich, ReagentPlus ≥99.0%), and sodium chloride (NaCl) (EMD chemicals, GR ACS crystals) in deionized water (18 MΩ·cm). Three water samples have been prepared with 0.1×, 1×, and 10× levels of contaminant concentration limit in water, respectively. The DIS platform measurements, along with comparative Solartron impedance analyzer measurements, have been obtained at 20 °C room temperature, at 200 mV AC amplitude, at the AC frequency range from 30 kHz to 55 kHz, and are shown in Figure 7. Overall, good agreement in impedance measurements was obtained between the DIS platform and the Solartron, to within a ±15% comparative error level in impedance modulus, and the DIS platform signal-to-noise response was sufficiently high to allow a reliable differentiation between varying contaminant concentration levels in water, either below or above the quality limit set by state-level agencies. Although contaminants in water may come with many different ions and with varying relative concentrations, such a DIS platform can be used, for example, as a non-selective distributed sensor for early detections of runoffs from agricultural/industrial activities, where sudden impedance anomalies in water can be detected within seconds and provide a trigger for emergency measures.

### 3.3. Glass Touchscreen Sensors

The development of glass compounds that transmit both light and electricity represents one of many challenges in the field of materials sciences. Phosphate-based glasses, depending on the glass composition, exhibit high solubility to metallic ions, high chemical and mechanical stability, and high glass transition and crystallization temperatures [20]; in particular, the phosphate glasses belonging to the AgI-AgPO_3_-WO_3_ system provide high AC ionic conductivities at room temperature (*i.e.*, 10^−2^ ≥ σ ≥ 10^−3^ S·cm^−1^) at kHz driving frequencies. This set of attributes are of great interest to science and technology as these glasses may form the basis for electro-optics modulators for telecommunication devices where an electrical field applied to the solid can modulate the phase, the frequency, the amplitude, or the polarization of the incident light passing through the material [21,22]. They may also be used as microprobes to measure the electrical activity of neurons in the brain [23], or integrated into smart textiles to perform radio-frequency emission and electrical interconnect functions [24].

**Figure 8:**
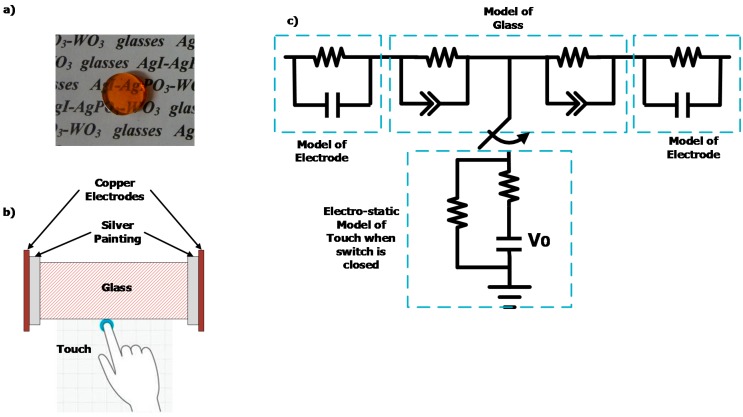
(**a**) Picture of the 45AgI-(55-*x*)AgPO_3_-*x*WO_3_ glass sample with *x* = 15 mol%; (**b**) Representation of the glass touchscreen setup, wherein the copper electrodes were connected to the DIS platform; and (**c**) equivalent electro-static circuit of the AgI-AgPO_3_-WO_3_ glass touchscreen.

In this work, the AgI-AgPO_3_-WO_3_ glass system [20] and the DIS platform have been used in tandem as the basis for a capacitive touch sensor. Many types of sensors use capacitive sensing, including interface devices, such as trackpads and touchscreens [25] that can replace mechanical buttons on screens, doors, and windows. These interface devices are generally enabled by the use of conductive specialty thin films (such as Indium-Tin-Oxide, ITO) that tend to be expensive due to containing elements like indium and due to requiring vacuum deposition processes. However, the use of AgI-AgPO_3_-WO_3_ glasses could open new opportunities for touchscreen devices as they can be manufactured in large float-glass quantities at low cost. For this study, 10 mm diameter, 8 mm thick, 45AgI-(55-*x*)AgPO_3_-*x*WO_3_ glass samples, with *x* = 0, 12, 15, and 20 mol%, have been used, as pictured in Figure 8a. Two copper electrodes were pasted on the side of each glass samples using silver paint; the silver paint used consisted of colloidal silver from Pelco^®^ with a sheet resistance of 0.02–0.05 ohms/sq/mil. Figure 8b,c illustrate the principle behind glass touchscreens: an object (e.g., finger, conductive stylus) touches the conductive glass and alters the electrical coupling between the two electrodes, creating a grounded path along the touch point, thus changing the overall impedance of the system as recorded through the electrodes. An equivalent electro-static model of the glass touchscreen is illustrated in Figure 8c, wherein the touch function actuates the grounded state of the glass system.

**Figure 9:**
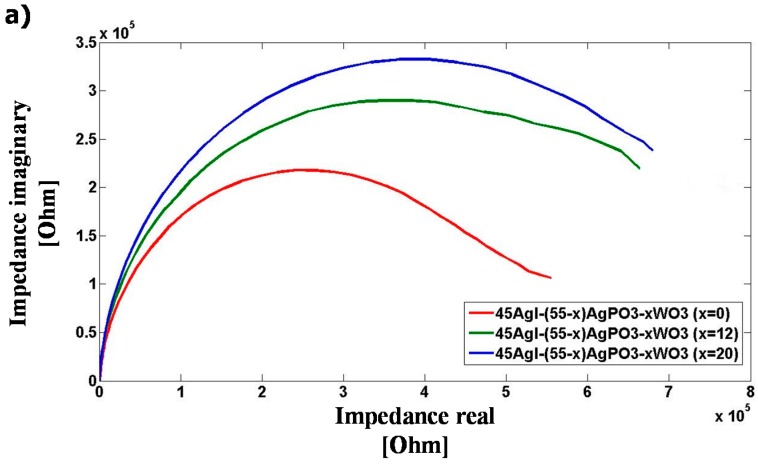

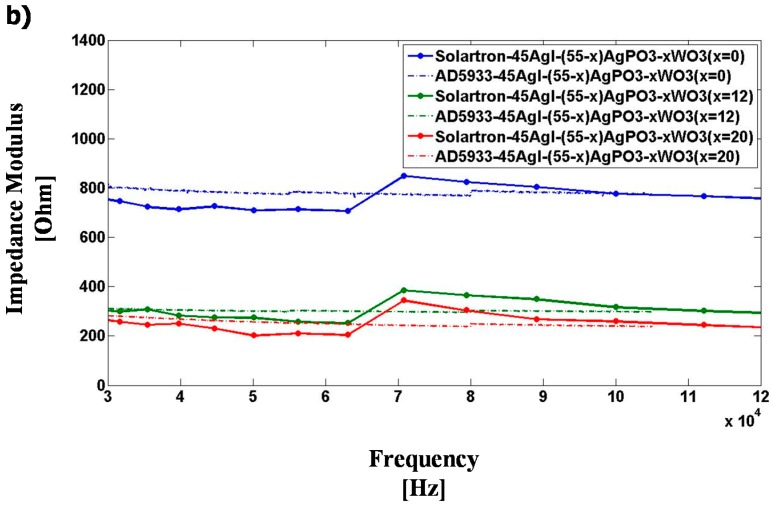
(**a**) Nyquist complex impedance of untouched 45AgI-(55-*x*)AgPO_3_-*x*WO_3_ glasses, using 200 mV excitation voltage on the Solartron Impedance analyzer from 1 Hz to 1 MHz frequency; and (**b**) Comparative impedance results obtained using the Solartron Impedance analyzer and the DIS Platform featuring an AD5933 device.

First, we have investigated the complex impedance of untouched AgI-AgPO_3_-WO_3_ glasses to characterize the materials under alternate current (AC). Figure 9a shows the Nyquist complex impedance spectra of 45AgI-(55-*x*)AgPO_3_-*x*WO_3_ glass samples with *x* = 0, 12, and 20 mol%. The impedance measurements were performed at 22 °C in ambient atmosphere using the Solartron Impedance analyzer. The measured Nyquist profiles are indicative of a resistance-capacitance parallel equivalent electrical circuit behavior [25] as illustrated in Figure 8c. The Solartron impedance analyzer measurements have been compared with the DIS platform measurements as shown in Figure 9b. Overall, good agreement in impedance measurements in the AC frequency range from 30 kHz to 120 kHz was obtained between the DIS platform and Solartron, to within a ±15% comparative error level in impedance modulus.

**Figure 10:**
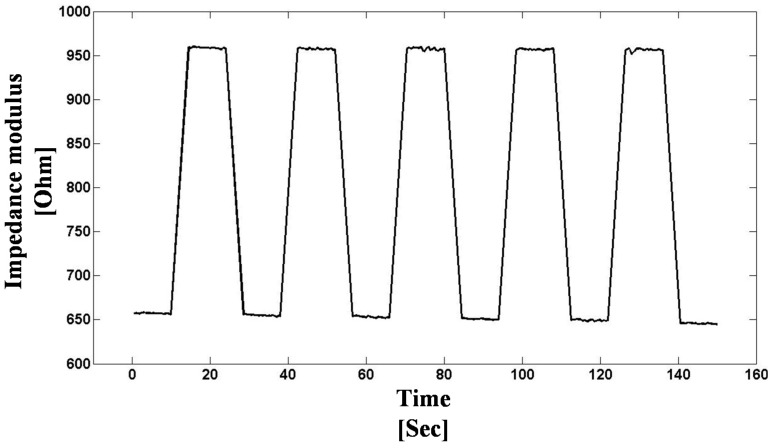
Touch sensing response of a 45AgI-(55-*x*)AgPO_3_-*x*WO_3_ (*x* = 15 mol%) glass sample recorded using the DIS platform at an AC frequency of 30 kHz, at 22 °C in ambient atmosphere. The time plot clearly resolves successive 12-s touches applied to the glass, with “touch” corresponding to higher impedance.

Second, we have investigated the impedance effect of a touched AgI-AgPO_3_-WO_3_ glass to characterize the materials as the basis of a glass touchscreen. Figure 10 shows the response of a 45AgI-(55-*x*)AgPO_3_-*x*WO_3_ (*x* = 15 mol%) glass touchscreen sensor recorded using the DIS platform at an AC frequency of 30 kHz. The DIS platform data acquisition is performed in less than 2 s, consequently the 12-s touches were clearly recorded, with a signal-to-noise ratio exceeding 50, thus demonstrating that the DIS platform can be used for in situ dielectric impedance applications pertaining to touch sensing, enabling advanced touchscreen applications like smart doors and smart windows without the use of expensive specialty thin film coatings on glass.

## 4. Conclusions

This paper described the development of a low-cost, miniaturized, multiplexed, and connected platform for dielectric impedance spectroscopy (DIS) designed for in situ measurements and adapted to wireless network architectures. The platform has been tested and used as a DIS sensor node on ZigBee mesh and was able to interface up to three DIS sensors at the same time and relay the information through the Internet for data analysis and storage. The system was built from low-cost commercial microelectronics components, performed continuous-frequency dielectric spectroscopy ranging from 5 kHz to 100 kHz, operated at 250 mW power consumption, and benefited from an on-the-fly calibration system that makes sensor calibration easy. The platform has been tested successfully for in situ dielectric impedance spectroscopy applications pertaining to fertilizer sensing, water quality sensing, and touch sensing, that find applications in many smart household, building, and industrial environments. To our knowledge, this is the first time a wireless platform featuring an AD5933 device was used along with touch and chemical sensors for environmental monitoring.
